# Potential of combining natural-derived antioxidants for improving broiler meat shelf-life — A review

**DOI:** 10.5713/ab.22.0188

**Published:** 2022-09-06

**Authors:** Andiswa Ntonhle Sithole, Vuyisa Andries Hlatini, Michael Chimonyo

**Affiliations:** 1 Animal and Poultry Science, School of Agricultural, Earth and Environmental Sciences, University of KwaZulu-Natal, P Bag X01 Scottsville 3209, Pietermaritzburg, South Africa; 2 Agricultural Research Council-Animal Production (Nutrition Building), Private Bag X2, Irene, 0062, South Africa; 3 Faculty of Science, Engineering and Agriculture, University of Venda Private Bag X5050 Thohoyandou 0950, South Africa

**Keywords:** Fatty Acids, Meat Waste, Microbiota, Oxidation, Reactive Oxygen Species, Synergy

## Abstract

Synthetic antioxidants have shown adverse effects on consumers. The review, thus, aims to assess the effect of marinating broiler meat with plant leaves-derived antioxidants potential for improving shelf-life and human health. Broiler meat loss and waste due to spoilage is more than three million kg annually, thus, extending shelf-life by reducing initial microbial load and autoxidation is essential. Adding various antioxidants would reduce oxidation of protein and fatty acids improving nutritional shelf-life through synergic interactions. Antioxidant synergetic effects also improves reduction in microbiota proliferation leading to the delayed development of off flavours and deterioration of meat colour. To reduce initial microbial load and autoxidation effects, the inclusion of polyphenols and antioxidants from varying sources by mixing various antioxidants would lead to improved synergic effects.

## INTRODUCTION

The increase in human population and the anticipated further increase incorporated with increase in households that earn higher income, thus, can afford to buy meat is forcing the meat industry to grow and produce meat rapidly [1]. The demand is weighing more on chicken meat as it is by far leading in consumption increase due to its affordability. In attempts to produce meat rapidly and meet profit margins, stocking density is increased from the welfare recommended 10 birds per m^2^ to more than 15 birds per m^2^ with projected 1.8 kg slaughter weights [2]. The increased stocking density assists with producing more meat per square metre, thus, greater chances of meeting profit margins. The increased stocking density, however, further deteriorates the relatively short broiler meat shelf life. Increased free radicals from greater reactive oxygen species (ROS) that oxidise the abundant polyunsaturated fatty acids (PUFA) [3,4]. To scavenge the free radical’s synthetic antioxidants are applied.

Synthetic antioxidants such as butylated hydroxy anisole, butylated hydroxy toluene, and synthetic tocopherol are being added to stabilize free radicals and delay nutrient oxidation. Adding synthetic antioxidants to reduce oxidative damage, however, may potentially cause adverse health effects and consumers prefer natural antioxidants, therefore, it remains a challenge to the meat industry [5,6]. In response to this challenge, research has shifted towards the investigation of natural antioxidants which are important in maintaining health [7]. Pre-harvested plant-derived antioxidant have been used to minimize oxidation in meat products [8], however, one source may not be sufficient in efficiently reducing oxidation. Polyphenols with assistance from amino acids may act as enhanced antioxidants, however, individually may not be efficient. Polyphenols may also enhance essential oils’ ability to reduce microorganisms. Varying flavonoids may disrupt microbiota membrane to inhibit the growth of microorganisms and chelate metal leading oxidation inhibition [9–11]. The use of multiple sources instead of one may, thus, yield improved meat shelf-life due to synergetic relationships amongst antioxidants that may render them superior to single ingredient antioxidants. Blending specific chemical compounds may lead to further synergetic action which further improves meat shelf-life [12,13].

Natural antioxidants sources include legumes, tree leaves, cereals, fruits, seeds, spices, vegetables, teas, and herbs [14,15]. Cereals, fruits, and vegetables form the greater part of a human diet, thus would create food-feed competition. Legumes, teas, and trees are rich in phenols, tannins, flavonoids, vitamins that have antioxidant properties and a minimized food-feed competition. High polyphenolic compound inclusion reduces growth performance in broilers before enough polyphenols are incorporated into the meat. The amount incorporated, however, is sufficient to reduce the effect of ROS at growth [16,17]. Marinating broiler meat with the leaf meal and leaf meal extracts may, therefore, provide a more concentrated form of polyphenols, thus, leading to more polyphenols in the meat [18,19].

Adding natural antioxidants may reduce the rapid oxidation of protein and extend nutritional shelf-life. The rapid alteration in visual appearance during storage would be reduced and an improved quality product would motivate consumers to trust and buy the meat [20]. The reduced shelf-life that is a cause for concern for meat retailers, wholesalers, and consumers while transitioning in the supply chain would be combated. Meat being partially spoiling whilst consumers are travelling home may be improved at disadvantaged communities along improved nutrients quantity of nutrients. The development of off flavours and deterioration of meat colour may also be delayed. This review, thus, aims to present and evaluate the potential of processing broiler meat with plant derived leaf meal antioxidants on improving shelf-life and human health.

## EFFECTS OF REDUCED BROILER MEAT SHELF-LIFE AND MEAT WASTE

The global meat industry is constantly evolving because of consumer preference change, lifestyle concerns, monetary, geographical, political, cultural, religious factors and food loss and waste [8]. Most variable loss and waste occur during handling, storage, processing, distribution, market, and consumption level depending on region [21]. Remarkably, farmers are reducing losses; however, it is evident that most developed countries lose more meat at consumption and developing countries loose meat as early as processing [22]. The loss in profits in developing countries is distributed amongst processors, wholesalers, and greatly consumers. This implies that exporting fresh produce for developing countries is not feasible, however, importing from developed countries is possible. This then leads to more loss in meat in developing countries as they cannot export chicken breasts that most developed countries prefer as fresh meat. Developing countries have made vacuum packed meat more expensive, thus, less people afford the meat leading to more retail loss. Methods such as meat salting, canning, fermentation, and smoking were also put to use to extend shelf life, however, may had not been effective enough [23].

The industry has added cold logistical chains, thermal treatment which includes super chilling, ultrarapid freezing, immersion vacuum cooling, pressure-shift freezing, dielectric heating and ohmic heating to extend shelf-life. In efforts to increase consumer safety, meat quality and nutritional shelf-life; traditional cutting and paper or waxed paper wrapping was replaced by store cutting and display on packages in fridges for self-service [23]. Packaging has also modified package atmosphere by vacuum ceiling, active packaging, and smart packaging [23]. These methods are, however, still challenged by the initial microbial load which hinder the preservation of meat nutrients at storage [24]. The other concern is skilled enough workers to handle the meat correctly. More than 3.5 billion kg per annum of poultry and meat is wasted at the consumer, retailer and foodservice levels and mainly attributed to microbial spoilage [25]. Increasing shelf-life could potentially save 5% of this meat and that could potentially feed more than 300,000 people. The added oxidative stress from heat stress and a greater stocking density also adds to the yet to be resolved shelf-life issue.

## POTENTIAL OF PLANT-DERIVED ANTIOXIDANTS ON EXTENDING MEAT SHELF-LIFE

Various literature has established the potential capacity of plant derived natural antioxidants to delay oxidation process and extend the shelf-life of meat products. Poultry producers have been encouraged to use natural antioxidant, such as, natural dietary alternatives and plant extracts to reduce the negative effect of lipid and protein oxidation. Plants extracts rich in polyphenol are known for several health benefits, examples being, *Punica gratun L* species which is rich in polyphenolic compounds and flavonoids reduces lipid oxidation in human bladder cells [26]. This necessitates research that focuses on using natural antioxidants from plant sources to replace synthetic antioxidants in the poultry industry. The effects of natural antioxidants, however, depend on various factors which include plants state when being utilized.

## EFFECT OF PRE- AND POST-HARVEST PLANT DERIVED ANTIOXIDANTS ON BROILER MEAT SHELF-LIFE

Plants amino acids and minerals are reduced as the plant ages, however, pre-harvest they are greater than after processing. Polyphenols increase as the plant ages pre-harvest, thus, the chemical component the plant is needed for is important. This also demonstrates that one plant may not be ideal for the best antioxidants’ composition and capability. The use of plants at a younger age may assist improve shelf-life by providing amino acids and minerals which may scavenge free radicals, such as, carbonyls leading to a greater meat colour, tenderness, juiciness, and off odour shelf-life. This, however, would not scavenge sufficient hydroxyls, peroxyls and alkyl radicals which ultimately lead to the carbonyls, hydroperoxides, hydrocarbons and alcohols produced by protein oxidation. Ultimately, the shelf-life of the meats’ nutritional value, flavour, tenderness, and colour is not efficiently extended. The consumers recurring purchase may also be reduced by the reduction in flavour, tenderness, and colour.

Polyphenols assist with hydrogen abstraction; their reduction may lead to a reduced hydrogen abstraction efficiency, thus, greater autoxidation and deteriorated fatty acid profile. Amino acids may have a weak antioxidant capability; however, amino acids improve polyphenols’ ability to scavenge on free radicals leading to an efficient oxidation reduction in meat fats and protein. Polyphenols and essential oils have a synergistic relationship that reduces microbial load which may ultimately extend shelf-life. The use of blended sources has been documented to be superior to individual sources, thus, fresh leaves may be difficult to incorporate [27]. Extracting fresh leaves has been documented to have lower antioxidant capacity compared to air dried plant material. The time of the year the plant is being harvested at is, however, also essential as harvesting during dry season may lead to plants with greater polyphenols than when harvested in a wet season. The expense in feeding natural antioxidants extracts may be high leading to a hike in production costs. Various antioxidant application methods for improved meat quality, therefore, need to be explored. Individual leaf meals influence few parameters, blending them may produce an improved shelf-life extension.

As plants dry, their antioxidant capacity is significantly reduced. There was a 6.4% loss in antioxidant capacity for celery vegetable plants dried by air and 33.7% for coriander dried by oven [28]. Some plants species show a positive correlation between antioxidant activity and vitamin C, vitamin E, and beta-carotene content [28]. The phenol, carotenoids, chlorophyll, and antioxidant capacity of fresh plants are higher than that of dried plants. A plant’s maturity stage affects its polyphenol content and overall quality. For instance, strawberry accumulates flavanols at high levels during early stages, whereas anthocynanins synthesis starts later and is most abundant when berries are ripe [29]. Karlund et al [29] found that total phenolics, total flavonoids, and total antioxidant activity were higher at the pink stage than at the ripe stage.

## POTENTIAL OF BLENDING PLANT ANTIOXIDANT SOURCES ON BROILER MEAT SHELF-LIFE

Lipid oxidation, protein oxidation, and meat spoilage bacteria are the main contributors to the reduction in the shelf-life of meat; thus, these leaf meal characteristics may be beneficial. Oxidation reduces the nutritional value of meat by deteriorating essential fatty acids, proteins leading to an unacceptable flavour, and toxins generation. Oxidation of meat in human leads to the nutrients losing its function causing disorders, such as, cancer, diabetes, obesity, dysfunction of red blood cells, and heart diseases. Bacteria, such as, *Pseudomonas* spp. and *Lactobacillus* spp. lead to off odour, discoloration, gas production, slime production and pH reduction [30]. Application of antioxidants and antimicrobials maintains the quality and safety of meat while also extending shelf-life and spoilage time [31,32]. Plant based antioxidants, however, need to be harvested and processed carefully to ensure high antioxidant capacity.

Blending of antioxidants sources may be superior as it would allow the use of various plant sources which assists with keeping the environmental resources at balance. Further, increases sources may increase chances of high radical affinity antioxidants threshold is met to optimize antioxidation in meat. It is noteworthy that synergic relationships may occur between chemical components within a leaf meal and between leaf meals as most these relationships are chemically related. It is also, however, evident that minimal plants have sufficient within plant synergic relationships to efficiently improve meat quality and shelf-life.

The effects various antioxidant sources have on meat which is not limited to shelf-life, however, limited to parameters that determine shelf-life. Oreganos’ documented effect is its antibacterial activity which may increase shelf-life [33–35]. Rosemary stabilizes redness, reduces oxidation and enhanced meat flavour. Clove improves water holding capacity, reduces thiol groups and thiobarbituric acid reactive substances (TBARS) and increases redness. This, however, does not guarantee that the redness is stabilised as it is increased. Blending clove, rosemary and oregano may lead to greater shelf-life extension as they all have varying characteristics which all contribute to an extended shelf-life. These spices, however, may be limited to certain areas and costly at other areas.

Umsasane (*Vachellia tortilis*) (high flavonoid), Umunga (*Vachellia karroo*) (low flavonoids), Paperback thorn (*Vachellia sieberiana*), babul (*Vachellia nilotica*) (medium flavonoids), Moringa (*Moringa oliefera*), uMhlonyane (*Artemisia afra*) (medium flavonoids), Buffalo thorn, Umphafa (*Ziziphus mucronata*) (low flavonoids), Rooibos (*Aspalathus linearis*) (high flavonoids) are potential plants that may be blended. They have not been studied extensively, however; the minimal studies published have demonstrated their potential if blended as they have antimicrobial and antioxidant capabilities. Blending these various antioxidant sources may yield superior shelf-life compared to individual sources.

The increase in the various antioxidant sources may lead to synergetic effects and strategic selection of sources may also be advantageous [36]. Considering current use of the plant material and their availability; these plants have a potential to be produced at a large scale and are already readily available at a wide scale. They also have minimal or natural aroma, thus, may induce neglectable undesirable odour in the meat. The optimal concentrations at which the leaf meals should be included is difficult to estimate as their chemical composition is not well studied, and synergic effects are complicated and hard to predict. The method used to apply antioxidants may not be effective or lead to consumer negative response to products which is the challenge the industry is currently encountering. Blending antioxidants would ensure a sustainable environment and complement one another once desirable concentrations have been established.

### Potential and mechanism of blended antioxidant plant sources on microbiota and meat shelf life

Microbiota refers to the ecological communities of commensal, symbiotics and pathogenic microorganisms. When the chicken is slaughtered, the microorganisms on the knife used to slaughter, from the intestines, water used for defeathering, and surfaces of the abattoir end up on the carcass. These microorganisms then prolificate to reduce meat shelf life. Meat spoilage during storage is also believed to dominantly be due to the microbial action from microbial flora directly after glycogen is depleted and pH of above 5.8 is reached. Ammonia, amines, alkaline post-mortem auto-enzyme metabolism and microbiota metabolism products from amino acids degradation combined, ultimately, lead to higher pH during storage [37,38]. Lower pH and antioxidants may lead to the disruption in microbiota proliferation reducing these effects.

### Significance of blending microbiota reducing antioxidants on meats’ sensory attributes

At low temperature *Pseudomonas s*pp., *Bronchothrix*, *flavabacterium*, *phytobacteria*, *Moraxella*, *staphylococcus*, *micrococcus*, *clostridium*, lactic acid bacteria and *Enterobacteriaceae* are encountered [39,40]. Flavonoids may disrupt microbiota membranes, chelate metal leading to the inhibition of microorganisms’ growth [9–11]. This is supported by the correlation between total phenolics and antibacterial activity [41]. The microbiota degraded protein may, however, not be restored which may reduce water holding capacity, increase drip and cooking loss. It is, therefore, essential to reduce initial microbial population by marinating. Murali et al [27] has demonstrated how blending chemical components from various sources is needed and should be done consciously. Moreover, finding the right concentration for varying sources has proven difficult as polyphenols may act as pro-oxidants at high concentrations essentializing testing at varying concentrations. Further, varying polyphenols have varying roles in inhibiting microbial growth which range from disputing cell membranes, inactivating enzymes, and binding to proteins.

### The blended antioxidant synergy on microbiota management

Reduced pH coupled with phenolic compounds increase the hydrophobicity of essential oils increasing essential oils affinity to pathogen attachment. Essential oils attachment and penetration of the pathogens’ membrane terminates these pathogens reducing their ability to prolificate. Catechins and cinnamic acids, however, disrupt cell membranes, inhibit bacterial toxin activity by acting on the protein and not the cell [42]. Tannins are said to bind proteins or inhibit microbial enzymes and flavones inactivate microbial enzymes. Flavonoids, such as, hesperidin, hesperetin, gallic, caffeic, and protocatechuic acid have anti-infective and anti-replicative effects on microbes [43,44]. Hydrophobic phenolic compounds are also bound by the microbial lipid bilayer leading to destabilized microbial cell walls solidifying cell wall and intracellular organelles ultimately inhibiting intracellular enzymes. Solidified cell contents reduce the amount of slime responsible for further pH decline during storage, thus, further reducing chances of microbial proliferation.

### Potential leaf meals that may be blended for optimum microbiota management

A single leaf meal meeting all these characteristics has not been established, nor has a combination of leaf meals and their concentrations. The combination of the various antioxidants and essential oils are essential in marinade raw materials chosen. Their synergic relationship is, however, not well understood, thus, blending *V. tortilis*, *V. tortilis*, *A. afra*, *A. nilotica*, *Z. mucronate*, and rooibos may lead to better understanding of synergistic action which may significantly reduce microbes on meat [12]. It is, therefore, essential to explain synergy using chemical composition and relationships to ensure an enhanced understanding of the current topic. This way, analysing leaf meals may yield a reasonable synergy estimation. It is evident that reducing initial microbial population with marinade may be beneficial, however, there is an indication that synergetic relationships between polyphenols being complicated [27,45].

### Synergetic effects that extend multiple parameters shelf-life by microbiota management

Various microbiota populations are reduced by varying polyphenols. There is, therefore, a great need for the marination of meat with polyphenolic compounds sources with varying types of polyphenol and concentrations. Reducing microbial population that utilizes nutrients from the meat to prolificate and release volatile organic compounds would also increase nutritional value and sensory attributes shelf-life [14]. Marinating with varying polyphenolic compounds sources may, therefore, lead to a lower pH, reduced microbial accumulation at storage, thus, reduced microbial related protein denaturation. The reduced denaturation of protein will increase protein bound water, thus, reducing drip loss. The interaction between proteins and polyphenols form bonds between amines and phenolic groups and could alter surface protein charges increasing the meats’ ability to retain water resulting in succulent meat at longer storage intervals [46]. The increase in meats ability to retain water would lead to an increase in consumers’ meat acceptance even at longer storage intervals.

A correlation between antimicrobial and antioxidant activity has not been established, thus, oxidation should not be neglected as nutrient’s oxidation affects the nutritional composition of meat reducing shelf-life. There is, however, a documented positive correlation between lipid oxidation, protein oxidation and water holding capacity [47]. Blending antioxidants that reduce both lipid and protein oxidation may lead to an improved water holding capacity shelf-life of meat. Reducing oxidation may, therefore, assist improve the water holding capacity of meat during longer storages [47].

### Potential of blended antioxidant plant sources on reducing nutrient oxidation and extending meat shelf-life

Oxidation is the loss of electrons by a molecule, in this case the molecule being proteins and PUFAs. The loss of electrons leads to nutrients losing its function [47]. The nutrients most susceptible to oxidation are lipids and proteins due to their chemical structure. Antioxidants may, therefore, reduce meat texture, increase flavour and colour resulting in better sensorial quality. Protein oxidation is initiated by secondary products of lipid oxidation, such as, hydroperoxides, superoxide anions, and hydroxyl radicals. The oxidants oxidise amino acids, dominantly sulphur containing amino acids, such as, cysteine and methionine. The presence of these amino acids in marinade may, therefore, assist reduce oxidation.

Adding viable substrates, such as, lysine, proline, arginine, and histidine to interact with aldehydes instead of meat amino acids interacting to form carbonyls. The greatest amino acid profile is obtained with immature plants; thus, immature plant sources would lead to improved results. Polyphenols and amino acids may reduce protein oxidation ultimately amino acid residues resulting in the reduced loss of essential meat amino acids. This demonstrates the importance of other nutrients that have synergetic effects on other antioxidants that have not attracted attention. There should, therefore, be consideration of extracting a mixture of nutrients rather than focusing on phenols alone to ensure nutritional composition is preserved the best way.

Proteins, such as, myoglobin are oxidized by oxygen into oxymyoglobin. When a ferrous molecule reacts with oxymyoglobin they form ferrous-oxymyoglobin. When antioxidants, such as, vitamin C, E, selenium, calcium, magnesium, cysteine, methionine, flavonoids, tannic acid and phenolic acid are added, ferrous-oxymyoglobin from being oxidized to ferric metmyoglobin. This ensures meat maintains colour stability longer during storage by preventing the initiation of lipid peroxidation and protein oxidation. Protein bound water is also, therefore, maintained leading to tender, succulent and flavourful meat as minimal nutrients may be lost due to improved water holding capacity during storage. Superoxide anions and hydrogen peroxide production may also be reduced, reducing the production of reactive hydroxyl radicals that can initiate lipid and protein oxidation.

Lipid oxidation is initiated by light and transition metals (Cu and Fe) that activate oxygen to ROS, such as, hydrogen peroxide (forms hydroxyls) and superoxide.Antioxidants, such as, vitamin C, E, selenium, calcium, magnesium, cysteine, methionine, flavonoids, tannic acid and phenolic acid scavenge on the ROS and have various affinity to various ROS. The incorporation of multiple sources of antioxidants will supply substrate for activated species reducing free radicals that may abstract hydrogen from unsaturated fatty acids. Blending rooibos, *V. tortilis*, *V. nilotica*, *Artemisia afra*, *Ziziphus mucronate*, and *V. karroo* may donate hydrogens efficiently to the free radicals. Alkyl radicals formed may reduce and may be further reduced by hydrogens donated from these species before they react with oxygen to form peroxy radicals [48,49]. The hindrance of highly reactive peroxy radicals by antioxidants reduces hydrogen abstraction from PUFAs limiting the formation of hydrogen peroxide and alkyl radicals [25]. This also reduces aldehydes, ketones, acids, and alcohols which are some of the alkyl radicals that change flavour, aroma, taste, and nutritional value in meat during storage [50]. In most of these studies shelf-life is, however, not determined, demonstrating the need to determine shelf-life effects on some of these species.

Rooibos, *V. tortilis*, *V. nilotica*, *Artemisia afra*, *Ziziphus mucronate*, and *V. karroo* synergic relationship is not well understood, thus, blending them may lead to better understanding of synergistic action of leaf meals which improves meats stability. The high quantity of PUFAs ensured by antioxidants ensures essential fatty acids are also maintained improving life expectancy, immune systems, and healthier brains especially for developing children.

### Importance of reduced oxidation on the fatty acid profile to human health

Essential fatty acids are omega 3, omega 6, omega 9 acids and saturated fatty acids in relation to the total fatty acids. Omega 3, omega 6 arachidonic, eicosapentaenoic acid are regarded as inflammatory markers which is why their ratio is essential to study [51]. Only alpha-linolenic acid, an omega-3 fatty acid and linoleic acid an omega-6 fatty acid are, however, known to be essential for humans [52]. The optimal desired ratio is n-6 to n-3 fatty acids in the human diet is 10.1 to 5.1 [53], the bracket of the ratio has since been reduced to 9.1 to 10.1 [54]. Vitamin E, a-tocopherol, are the major lipid-soluble antioxidant in animal tissues which may act post-mortem to insure unsaturated fatty acids are not converted to saturated fatty acids beyond the desired ratio [55,56]. Oxidation that manifests as a conversion of the red muscle pigment myoglobin to brown metmyoglobin may be reduced by carbon, hydrogen and multiple double bonds containing molecules. Adding antioxidants may, therefore, reduce the development of rancid odours and flavours from the degradation of the PUFAs in the tissue membranes and overall shelf-life. Further, hindering lipid oxidation reduces protein oxidation as lipid oxidation products lead to protein oxidation.

Reducing oxidation in meat samples by using antioxidants stabilised meat colour during storage. Some leaf meals however may not have sufficient antioxidizing power leading to more yellowness and lightness appearance, such as, garlic leading to consumer reduced acceptability. Due to the oxidised protein that leads to light being scattered making meat appear lighter. Shear force may also be increased due to antioxidant sources that do not have sufficient antioxidizing power leading to protein oxidation, thus, lower water holding capability along higher drip loss. To improve oxidizing power synergy may be essential for blended plants.

## SUMMARY

The addition of polyphenols in efforts to improve broiler meat quality and increase meat shelf-life should be explored more to reduce loss/waste of meat. Using polyphenols as marinade may increase broiler meat shelf-life by at least 3 days which may be longer with varying antioxidant sources. Feeding and marinating the meat may be advisable as feeding is a first line of defence during rearing and when enzymes are defeated nutritional components and antioxidants may be of assistance. Meat shelf-life parameters, such as, redness are hard to extend as the metmyoglobin formation delay has not been well studied. The inclusion of medicinal plants may, however, have an added advantages as they may prevent or reduce humans’ susceptibility to a wide range of sickness. Marinating broiler meat with medicinal plants which are not widely used may assist maintain biodiversity and combat sickness in humans. It is, therefore, advisable to use these hardly used bush encroaching plants for human benefits. More studies, however, need to be conducted to determine which antioxidant combination works better on which parameters and/or microbes. An extensive chemical composition of polyphenols especially of *V. karroo*, *V. nolitica*, *Artemisia afra*, *Ziziphus mucronate*, *V. tortilis* and rooibos need to be explored to make it easier to extrapolate how they may affect meat shelf-life once polyphenol combination is established. Concentrations needed of these leaf meals and of individual polyphenols to be combined are also essential and need more attention.
